# Digital gene expression profiling analysis and its application in the identification of genes associated with improved response to neoadjuvant chemotherapy in breast cancer

**DOI:** 10.1186/s12957-018-1380-z

**Published:** 2018-04-23

**Authors:** Xiaozhen Liu, Gan Jin, Jiacheng Qian, Hongjian Yang, Hongchao Tang, Xuli Meng, Yongfeng Li

**Affiliations:** 1 Pathology Department, Zhejiang Cancer Hospital, Hangzhou, 3110022 Zhejiang Province China; 2 The 2nd Clinical Medical College, Zhejiang Chinese Medical University, Hangzhou, 310053 Zhejiang Province China; 3 Department of Breast Surgery, Zhejiang Cancer Hospital, Building NO. 1, East of Banshan Road, Gongshu District, Hangzhou, 3110022 Zhejiang Province China; 4 Department of General Surgery, Tongde Hospital of Zhejiang Province, Hangzhou, 310012 China

**Keywords:** Breast cancer, Digital gene expression, Neoadjuvant chemotherapy, Ubiquitin proteasome, Cytokine–cytokine receptor interactions

## Abstract

**Background:**

This study aimed to screen sensitive biomarkers for the efficacy evaluation of neoadjuvant chemotherapy in breast cancer.

**Methods:**

In this study, Illumina digital gene expression sequencing technology was applied and differentially expressed genes (DEGs) between patients presenting pathological complete response (pCR) and non-pathological complete response (NpCR) were identified. Further, gene ontology and Kyoto Encyclopedia of Genes and Genomes (KEGG) pathway enrichment analysis were then performed. The genes in significant enriched pathways were finally quantified by quantitative real-time PCR (qRT-PCR) to confirm that they were differentially expressed. Additionally, GSE23988 from Gene Expression Omnibus database was used as the validation dataset to confirm the DEGs.

**Results:**

After removing the low-quality reads, 715 DEGs were finally detected. After mapping to KEGG pathways, 10 DEGs belonging to the ubiquitin proteasome pathway (*HECTD3*, *PSMB10*, *UBD*, *UBE2C*, and *UBE2S*) and cytokine–cytokine receptor interactions (*CCL2*, *CCR1*, *CXCL10*, *CXCL11*, and *IL2RG*) were selected for further analysis. These 10 genes were finally quantified by qRT-PCR to confirm that they were differentially expressed (the log_2_ fold changes of selected genes were − 5.34, 7.81, 6.88, 5.74, 3.11, 19.58, 8.73, 8.88, 7.42, and 34.61 for *HECTD3*, *PSMB10*, *UBD*, *UBE2C*, *UBE2S*, *CCL2*, *CCR1*, *CXCL10*, *CXCL11*, and *IL2RG*, respectively). Moreover, 53 common genes were confirmed by the validation dataset, including downregulated *UBE2C* and *UBE2S*.

**Conclusion:**

Our results suggested that these 10 genes belonging to these two pathways might be useful as sensitive biomarkers for the efficacy evaluation of neoadjuvant chemotherapy in breast cancer.

## Background

Breast cancer is one of the most common malignancies, with the highest incidence in females among all cancers. Recently, neoadjuvant chemotherapy has attracted significant attention as a new treatment for patients with early and/or locally advanced breast cancer [1, 2]. It can reduce the size of the primary tumour, thereby conferring an operable status on a substantial proportion of patients with advanced tumours that were previously considered unresectable. In addition, this treatment helps patients become eligible for breast-conserving surgery and avoid mastectomy. It is well established that a pathological complete response (pCR) serves as an intermediate marker of a better long-term survival [3, 4]. According to postoperative pathological report, pCR is defined as the absence of invasive tumour cells in the surgical specimens of axillary lymph node and the primary lesion of breast cancer.

Nevertheless, neoadjuvant chemotherapy is not beneficial for all patients. Identification of predictive factors of neoadjuvant chemotherapy response would therefore be of great value to patients as these will help avoid side effects and unnecessary expenses. To date, the identification of clinical parameters for the prediction of pCR, such as tumour size, tumour grade, histology, and lymph node status, has been widely reported [3]. Additionally, hormone receptor, human epidermal growth factor receptor 2 (*HER2*), and Ki-67 [5, 6] have been extensively studied and are shown to be associated with pCR. However, the accuracy of these clinical and molecular parameters remains unsatisfactory. Thus, more accurate and clinically useful predictive factors need to be developed.

Recent evidence suggests that some genes involved in certain pathways may be important predictors of the neoadjuvant chemotherapy response. Witkewicz et al. discovered that deregulation of the retinoblastoma tumour suppressor pathway is associated with improved response to neoadjuvant chemotherapy [7]. Other studies also reported that the peroxisome proliferator-activated receptor signalling pathway plays an important role in the mechanism of action of neoadjuvant chemotherapy [8].

In this study, pathways susceptible to neoadjuvant chemotherapy were investigated in detail. Digital gene expression sequencing (DGE-seq) [9, 10] is a sensitive method that is useful for developing and refining the molecular taxonomy of breast cancer as well as investigating molecular heterogeneity [11]. Using this technology complemented with a novel, powerful, analytical method, we compared gene expression profiles of samples from patients presenting pCR with those of samples from patients with non-pathological complete response (NpCR). This study was designed to identify gene groups that could be used to distinguish primary breast cancers that are sensitive to neoadjuvant chemotherapy from those that are resistant to it and to identify the molecular pathways involved in the mechanism of action of neoadjuvant chemotherapy.

## Methods

### Sample collection

All pre-chemotherapy samples were collected from the tumour bank at Zhejiang Cancer Hospital, Hangzhou, China, using standard procedures. Before neoadjuvant chemotherapy, all patients underwent a tumour biopsy with a vacuum-assisted core biopsy instrument (Mammotome 8G; HH Ethicon Endosurgery/Johnson and Johnson Company, Langhorne, PA, USA) with ultrasonographic guidance for histological examination and gene expression analysis. Patients were treated with one cycle of docetaxel at 120 mg/m^2^ and epirubicin at 100 mg/m^2^, followed by four cycles of cyclophosphamide at 700 mg/kg. This study was approved by the Zhejiang Provincial Experimental Animal Management Committee, which has the authority to approve studies involving human samples under Contract 2014-3039 (ZEAC 2014-3039). Additionally, all patients provided written informed consent prior to the beginning of this study and were provided with an explanation of the principles of privacy of information that prevailed in this study. Twenty fine-needle aspirate (FNA) biopsies from 7 patients with pCR and 13 patients without pCR were collected.

### RNA extraction

RNA was extracted from FNA biopsy samples using the E.Z.N.A.™ DNA/RNA/Protein Isolation Kit (Omega, CA, USA), in accordance with the manufacturer’s instructions. The RNA pellet was dissolved in diethylpyrocarbonate (DEPC) H_2_O. RNA concentration and sample quality were assessed with a Nanodrop (ND2000 Spectrophotometer; Thermo Scientific, Wilmington, DE, USA). Samples were considered adequate for further analysis if the optical density 260/280 ratio was ≥1.8. RNA samples were stored at − 80 °C until use.

### Profiling library preparation for DGE-seq

Ten micrograms of total RNA from pooled RNA samples, including three pCR and NpCR samples each, was used for the digital gene expression profiling sequencing. In accordance with the manufacturer’s instructions, total RNA was purified using oligo-dT magnetic beads to yield poly(A+) mRNA and subsequently fragmented into short sequences in the presence of sodium hydroxide. Sequence library construction was performed in accordance with the instructions of the ScriptSeq™ mRNA-Seq Library Preparation Kit (Illumina®-compatible; Illumina, San Diego, CA, USA). Briefly, the fragmented RNA was reverse-transcribed into cDNA using the SuperScript Double-stranded cDNA Synthesis kit (Invitrogen, Carlsbad, CA, USA) with the addition of SuperScript III reverse transcriptase (Invitrogen) and random primers with a tagging sequence at the 3′ ends. This procedure was followed by RNase A (Roche, Basel, Switzerland) treatment, phenol–chloroform extraction, and ethanol precipitation. The 5′ DNA/DNA adaptor was ligated to the resulting cDNAs, and the di-tagged cDNA was purified with polyacrylamide gel electrophoresis (PAGE) gel. The insert fragment size was approximately 150–250 bp. The resulting sequences were PCR-amplified for 18 cycles using a high-fidelity DNA polymerase, and the products were purified on a 6% Tris/Borate/EDTA PAGE gel. DGE libraries were sequenced using a single flow cell on an Illumina Hiseq2000.

### Identification and functional analysis of differentially expressed genes

Next, DGE-seq results were compared between the pCR and non-pCR groups to identify changes in gene expression. The false discovery rate was used to determine the critical *P* value in multiple tests. We used a *P* value of ≤ 0.05 and an absolute value of the log2 (fold change) > 1 as the thresholds to identify significant differences in gene expression [12, 13]. We aimed to identify changes in gene expression between pCR and NpCR samples and to determine the molecular pathways transcriptionally affected by these changes. The fold change between reads of sequenced genes was used to identify genes with statistically significant changes in expression [14].

To characterise the functional consequences of changes in gene expression, Gene Ontology (GO) analysis, which provides a coherent annotation of differentially expressed gene (DEG) products, and pathway analysis of the DEGs, based on the Kyoto Encyclopedia of Genes and Genomes (KEGG) database, were performed [10].

### Gene quantification by quantitative real-time PCR

mRNA expression levels of the 10 selected candidate genes were validated by reverse transcription PCR (RT-PCR). Gene-specific primers were designed using Primer 5.0 software (data not shown). Each reaction was performed in a final volume of 10 μL containing 1 μg of total RNA, 1 μL of random primer (10 μM), 2 μL of 5× M-MLV buffer, 1 μL of dNTPs (10 mM; Takara, Tokyo, Japan), 0.5 μL of M-MLV reverse transcriptase (Takara), 0.5 μL of RNase inhibitor (Takara), and DEPC H_2_O. The mixture was incubated at 42 °C for 60 min and then at 70 °C for 15 min. All reverse transcription reactions were performed in a PCR S1000 Thermocycler (Bio-Rad, Hercules, CA, USA).

Gene quantification was performed using an SYBR green quantitative real-time PCR (qRT-PCR) array. qRT-PCR analysis was performed on an ABI PRISM 7500 Real-Time System (Applied Biosystems, Foster City, CA, USA) with 20-μL reaction volumes containing 1 μL of reverse transcription product as a template, 10 μL of Platinum SYBR Green qPCR SuperMix-UDG (Invitrogen, C11744-100), 0.4 μL of forward primer (10 μM), 0.4 μL of reverse primer, and DEPC H_2_O. The reactions were performed in 96-well plates at 50 °C for 2 min, 95 °C for 5 min, 40 cycles of 95 °C for 15 s, 60 °C for 31 s, and then 60–95 °C to obtain the melting curve. β-actin was stably expressed in the tissue and has been widely used as a standard control for normalisation [15]. Thus, it was also used as the reference gene in this study. For all genes, triplicate analyses were completed. After the run, amplification dissociation curves were assessed to exclude primer–dimer amplification. The relative expression of genes was calculated using the fold change of gene expression method. The fold changes of gene expression in each pCR sample relative to the expression in the NpCR sample were calculated using the following formula [16]:

$$ {}^{\Delta \Delta}\mathrm{CT}={\left({\mathrm{CT}}_{\left(\mathrm{gene}\right)}-{\mathrm{CT}}_{\left(\upbeta -\mathrm{Actin}\right)}\right)}_{\mathrm{pCR}}-{\left({\mathrm{CT}}_{\left(\mathrm{gene}\right)}-{\mathrm{CT}}_{\left(\upbeta -\mathrm{Actin}\right)}\right)}_{\mathrm{NpCR}} $$

The cycle threshold (CT) is defined as the number of cycles required for the fluorescent signal to cross the threshold in qRT-PCR [15].

### Validation of DEGs

To confirm the differential expression of the screened DEGs, the validation dataset GSE23988 was downloaded from Gene Expression Omnibus (GEO, https://www.ncbi.nlm.nih.gov/geo/) database. There were 41 residual disease (RD) samples and 20 pCR samples under GSE23988. Using limma package (http://www.bioconductor.org/packages/2.9/bioc/html/limma.html) [17] in R, the DEGs in RD vs. pCR comparison group were analysed. The *P* value < 0.05 was set as the threshold.

## Results

### Analysis of DGE-seq results

**Fig. 1:**
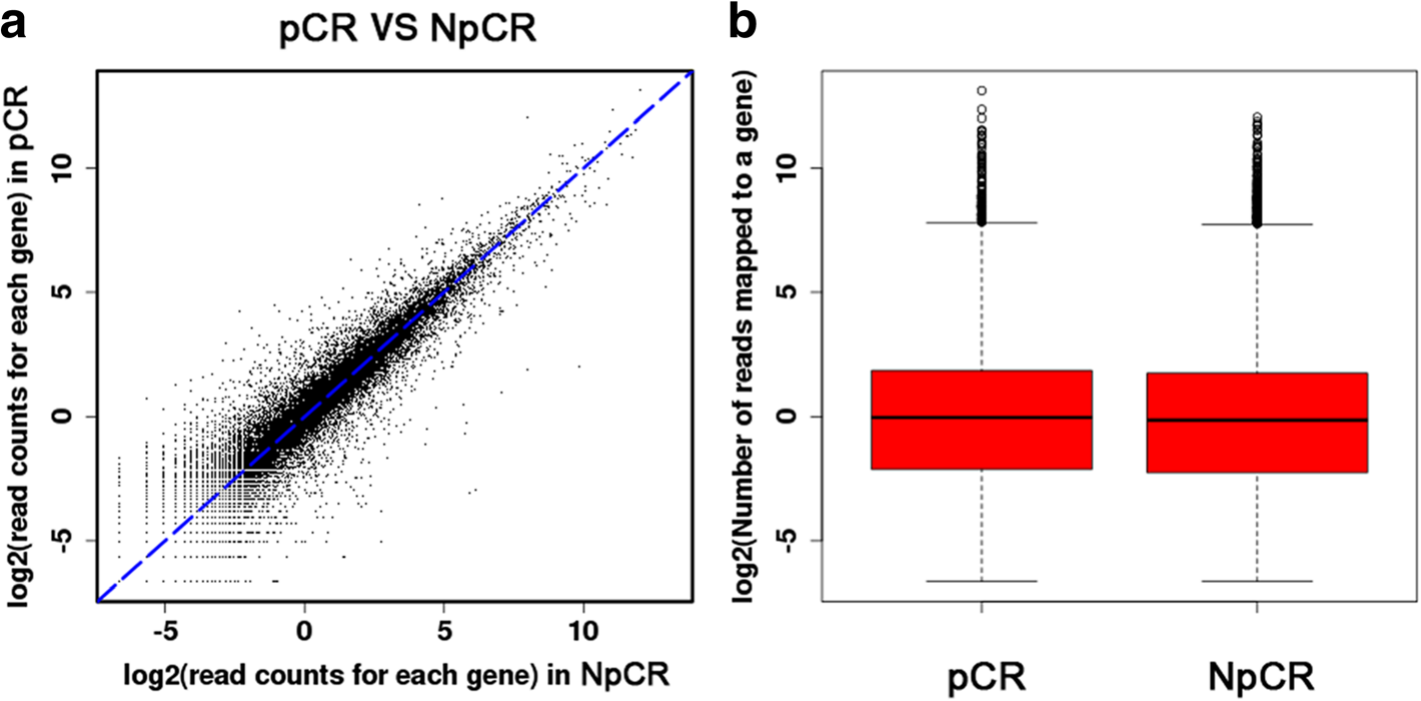
Gene expression distribution between pathological complete response (pCR) and non-pathological complete response (NpCR) samples. Summary of the digital gene expression sequencing (DGE-seq) mapping data. **a** A scatter plot comparing the gene expression levels between pCR and NpCR samples. **b** A box plot for the number of reads uniquely mapped to a transcript

In the key gene screening step, DGE-seq based on gene expression profiles was performed to identify genes from tissues obtained from patients presenting pCR and NpCR that were differentially expressed between these two groups. To gain insight into the transcriptome relevant to breast cancer, we used the Illumina Hiseq2000 platform to perform high-throughput DGE-seq [9, 11, 18] analysis on poly(A)-enriched RNAs from six breast cancer libraries, namely, NpCR1, NpCR2, NpCR3, pCR1, pCR2, and pCR3. Images generated by the sequencer were converted into nucleotide sequences using a base-calling pipeline. The raw reads were saved in the FASTQ format, and low-quality reads were removed prior to analysing the data, as previously reported [19]. After removal of the low-quality reads, we obtained a total of 10,690,546; 6,365,594; 5,792,665; 8,402,125; 8,830,451; and 6,619,228 clean reads from the libraries NpCR1, NpCR2, NpCR3, pCR1, pCR2, and pCR3, respectively. All subsequent analyses were based on the clean reads. The high-quality reads were selected and exclusively used in the mapping using TopHat. No more than two mismatches were allowed in the alignment for each read, and unique mapping reads were used in the latter analysis. DGE-seq data are summarised in Fig. 1.

### Identifying DEGs

**Fig. 2:**
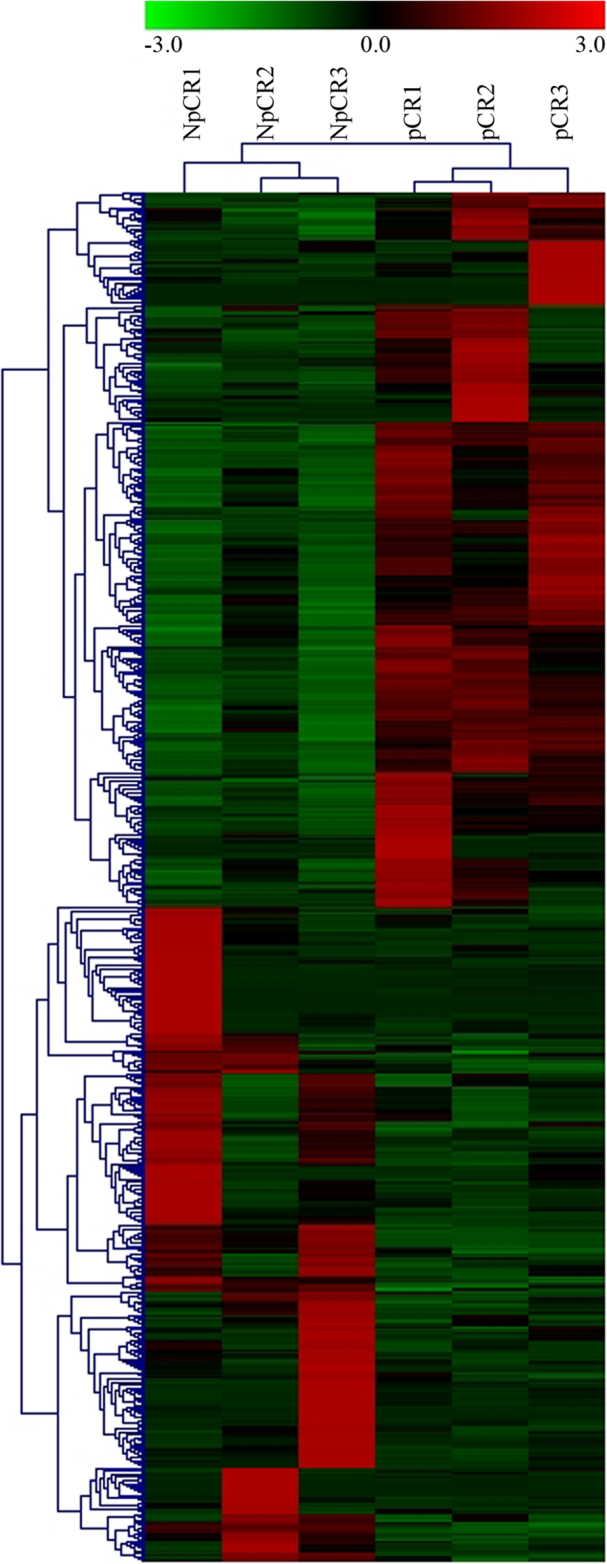
Cluster analysis of gene expression based on digital gene expression sequencing (DGE-seq) data. The heat map reveals the results from the cluster analysis of the DGE-seq data. Each column represents one of the 715 differentially expressed genes identified in our study. Each row represents a sample. For each gene, red indicates a high level of expression relative to the mean, whereas green indicates a low level. The scale bar below indicates the number of standard deviations from the mean

Using a twofold difference in expression as the cut-off level, we identified 715 genes that were differentially expressed between pCR and NpCR samples (Fig. 2). Among the 715 DEGs, 342 were upregulated in NpCR samples, whereas 373 were downregulated in them (data not shown).

**Fig. 3:**
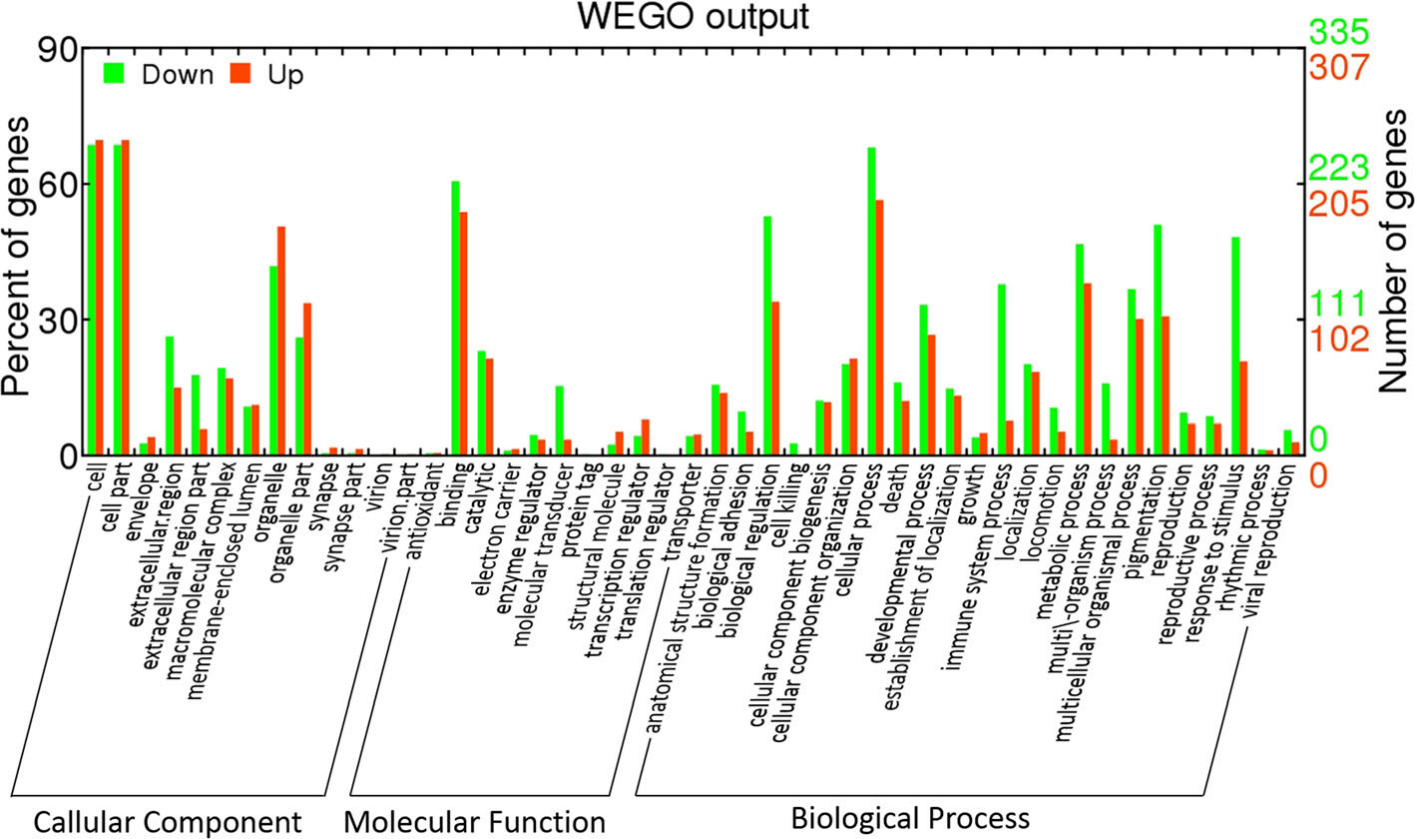
Histogram of the Gene Ontology (GO) functional analysis for the differentially expressed genes obtained from the digital gene expression sequencing (DGE-seq) data. The frequency of the GO terms was analysed using the Web Gene Ontology Annotation Plot (WEGO) method. The results are summarised in the following three main categories: (1) cellular component, (2) molecular function, and (3) biological process. The right *y* axis indicates the number of genes in a category. The left *y* axis indicates the percentage of a specific category type of gene in that category. Red indicates increased expression in the pathological complete response (pCR) group, while green indicates reduced expression in that group

GO and KEGG [20] were used to identify the functional categories and pathways that were particularly associated with the DEGs. The 715 genes were categorised into the three main GO classification categories (biological process, cellular component, and molecular function). They were particularly associated with the subcategories of ‘regulation of biological process’, ‘binding and catalytic activity’, and ‘metabolic process’ (Fig. 3).

The 715 genes were also mapped to the reference canonical pathways using KEGG. Genes upregulated in pCR samples were enriched for several pathways, including cytokine–cytokine receptor interactions [21, 22] and the ubiquitin proteasome pathway [23, 24]. In contrast, pathways involved in focal adhesion [25] and extracellular matrix (ECM) [26, 27] receptor interactions were enriched in NpCR samples. These findings are useful for investigating specific processes, functions, and pathways involved in breast cancer.

### DGE-seq data validation using qRT-PCR analysis

**Fig. 4:**
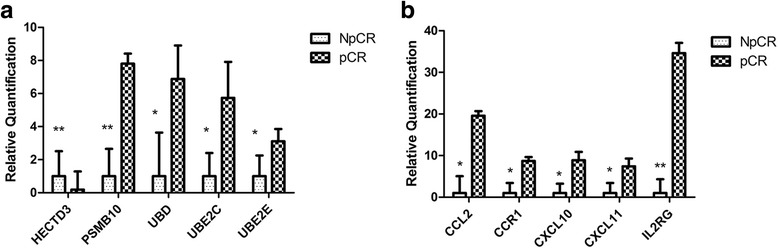
Characterisation of 10 differentially expressed genes by quantitative real-time PCR (qRT-PCR). **a** Expression of the ubiquitin proteasome pathway genes determined using qRT-PCR. Statistically significant induction of gene expression in pathological complete response (pCR) samples compared with that in non-pathological complete response (NpCR) samples is denoted by *P ≤ 0.05. **b** Expression of the cytokine–cytokine receptor interaction genes determined using qRT-PCR. Statistically significant induction of gene expression in pCR samples compared with that in the NpCR samples is denoted by *P ≤ 0.05

Considering its precise quantification and high sensitivity, qRT-PCR was used for a detailed analysis of the expression of these genes [28]. To investigate whether the ubiquitin proteasome and cytokine–cytokine receptor interaction pathways are sensitive to neoadjuvant chemotherapy, we measured the expression of genes involved in these pathways in tissues from pCR and NpCR samples. Twenty samples were analysed, including 7 from patients presenting pCR and 13 from patients presenting NpCR. qRT-PCR analysis of 10 representative genes, namely, five representative genes from the ubiquitin proteasome pathway (HECT domain E3 ubiquitin protein ligase 3, *HECTD3*; proteasome subunit beta 10, *PSMB10*; ubiquitin D, *UBD*; ubiquitin-conjugating enzyme E2C, *UBE2C*; and ubiquitin-conjugating enzyme E2S, *UBE2S*) and five genes from the cytokine–cytokine receptor interaction pathway (chemokine (C-C motif) ligand 2, *CCL2*; chemokine (C-C motif) receptor 1, *CCR1*; chemokine (C-X-C motif) ligand 10, *CXCL10*; chemokine (C-X-C motif) ligand 11, *CXCL11*; and interleukin 2 receptor, gamma, *IL2RG*), was conducted to verify the transcriptome sequencing expression profile data. Regarding the ubiquitin proteasome pathway, results indicated that the expression of *PSMB10*, *UBD*, *UBE2C*, and *UBE2S* was consistently upregulated more than twofold in samples from pCR patients compared with those from NpCR patients. In contrast, the expression level of *HECTD3* was lower in the group of pCR patients than in the group of NpCR patients (Fig. 4a). Regarding the cytokine–cytokine receptor interaction pathway, *CCL2*, *CCR1*, *CXCL10*, *CXCL11*, and *IL2RG* were significantly overexpressed in samples from pCR patients when compared with those from NpCR patients (Fig. 4b). The qRT-PCR analysis was consistent with the results obtained with the DGE-seq analysis.

### Validation of DEGs

Compared with pCR samples, a total of 1068 DEGs (506 upregulated and 562 downregulated) were obtained in the RD samples. There were 53 common genes between the 715 DEGs and the 1068 DEGs, including 26 upregulated genes and 27 downregulated genes (such as *UBE2C* and *UBE2S*).

## Discussion

The aim of this study was to assess gene expression differences between pCR and NpCR patients to identify patients who are sensitive to neoadjuvant chemotherapy. We conducted gene expression profiling using pre-chemotherapy FNA. The development of high-throughput deep sequencing technology provides almost complete views of DGE profiles. This highlights the potential of new large-scale parallel sequencing strategies to profile gene expression in malignant tumours [12]. The results provide experimental evidence supporting the authenticity of 715 genes found to be differentially expressed between pCR and NpCR patients, including 373 upregulated and 342 downregulated genes in pCR patients. Especially, *UBE2C* and *UBE2S* were confirmed to be significantly downregulated by the validation dataset GSE23988. Upon mapping the corresponding genes to biological pathways, various interesting observations emerged. For example, pathways involved in focal adhesion and ECM receptor interactions were particularly associated with NpCR samples, whereas genes involved in cytokine–cytokine receptor interactions, the ubiquitin proteasome pathway, and cell adhesion showed lower activity than those in pCR samples.

Two prominent pathways emerged from this analysis. Among the DEGs, those involved in cytokine–cytokine receptor interactions and the ubiquitin proteasome pathway were particularly prominent in pCR patients. In our analyses, we assessed biologically relevant gene pathways and categories rather than individual genes. Previous studies demonstrated that increased expression of an immune-related gene cluster is associated with the beneficial effects of neoadjuvant chemotherapy. Denkert et al. recently reported that the presence of tumour-associated lymphocytes in breast cancer is a new independent predictor of response to neoadjuvant chemotherapy [29]. Here, we observed that genes involved in cytokine–cytokine receptor interactions exhibited increased expression levels in samples from pCR patients. Cytokines act through receptors and are especially important in the immune system, health, and disease, specifically in the host response to cancer [21]. We believe that the host immune response enhances the ability of neoadjuvant chemotherapy to eliminate cancer cells. For example, mesenchymal stem cells (MSCs) may increase the population of breast cancer stem cells (CSCs) and promote the growth of breast tumour via generating cytokine networks [30, 31]. Enrichment analysis indicated that *CCL2*, *CCR1*, *CXCL10*, *CXCL11*, and *IL2RG* were enriched in cytokine–cytokine receptor interaction pathway. *CCL2*-induced chemokine cascade in macrophages contributes to the metastasis of breast cancer, and *CCR1* inhibition may be utilised to treat metastatic disease [32]. *CXCL10* may function as an algogenic molecule in the development of metastatic breast cancer-induced bone pain through spinal microglial activation [33, 34]. Overexpression of *IL-2* and its receptor chains (α, β, and γ) is correlated with breast cancer development and may also be related to the tumour malignancy [35]. Therefore, *CCL2*, *CCR1*, *CXCL10*, *CXCL11*, and *IL2RG* might be involved in neoadjuvant chemotherapy in breast cancer via the cytokine–cytokine receptor interaction pathway.

We also noted significant differential expression in a number of genes involved in the ubiquitin proteasome pathway. Targeting this pathway may thus serve as a mechanism of action for numerous anti-breast cancer agents. Proteolysis of a variety of proteins mediated by the ubiquitin proteasome pathway is a vital mechanism that regulates protein activity and function. The ubiquitin proteasome pathway is critical for cellular quality control and defence mechanisms, which are involved in numerous cellular physiological processes such as cell cycle regulation, division and differentiation, and DNA repair [36, 37]. Considering the large number of proteins and processes involved in this pathway, its aberrant regulation contributes to the pathogenesis of several human diseases. According to our results, *HECTD3*, *PSMB10*, *UBD*, *UBE2C*, and *UBE2S* were the representative genes from the ubiquitin proteasome pathway. Through regulating *HECTD3*, *miR-153* suppresses the survival of the patients with triple-negative breast cancer (TNBC) and acts as a potential tumour suppressor [38]. High expression of *PSMB7* indicates the shorter survival of breast cancer patients; therefore, *PSMB7* expression can serve as a poor prognostic marker in the disease [39]. *UBD* overexpression has an association with the epirubicin resistance of TNBC and predicts the adverse outcome of TNBC treatment [40]. Overexpressed *UBE2C* is related to worse survival of patients with breast cancer, indicating that *UBE2C* may play an oncogenic role in the progression of the disease [41]. *UBE2S* is correlated with the malignant characteristics (such as anchorage-independent growth, migration, and invasion) of breast cancer cells, and thus, *UBE2S* may be used as a therapeutic target for breast cancer [42, 43]. These suggested that *HECTD3*, *PSMB10*, *UBD*, *UBE2C*, and *UBE2S* might also affect the efficacy of neoadjuvant chemotherapy in breast cancer through the ubiquitin proteasome pathway.

Considering the small sample size of this study and the various neoadjuvant chemotherapies administered to these patients, these observations require confirmation by repeated observations and larger studies.

## Conclusions

In summary, 715 DEGs between pCR and NpCR samples were identified. Besides, *HECTD3*, *PSMB10*, *UBD*, *UBE2C*, and *UBE2S* involved in the ubiquitin proteasome pathway, as well as *CCL2*, *CCR1*, *CXCL10*, *CXCL11*, and *IL2RG* implicated in the cytokine–cytokine receptor interaction pathway, might be used for evaluating the efficacy of neoadjuvant chemotherapy in breast cancer.
